# Supplementary material for: Identification of a forkhead box protein transcriptional network induced in human neutrophils in response to inflammatory stimuli
Source: Front Immunol. 2023 Jan 20;14:1123344. doi: 10.3389/fimmu.2023.1123344 (PMC9900176; doi:10.3389/fimmu.2023.1123344)
Supplement: Supplementary file 1 [file DataSheet_1.pdf]

Study	GEO accession	Dose and time of LPS treatment	Platform	Number of biological replicates	Number of regulated genes (2-fold)
**Human**					
Our data	GSE221866	1 µg/ml 6h (in vitro)	Illumina NovaSeq 6000	3	2565 up<br>2457 down
Kotz et al	GSE22103	50 ng/ml 16h (in vitro)	Affymetrix Human Genome U133 Plus 2.0 Array	26 (burn trauma patients)	2119 up<br>2569 down
Khaenam et al	GSE49758	Final conc. 20% 6h (in vitro)	Illumina HumanHT-12 V4.0 expression beadchip	2	812 up<br>915 down
de Kleijn et al 2012	GSE35590	2ng/kg of body weight 2h (in vivo)	Affymetrix Human Exon 1.0 ST Array	4	461 up<br>343 down
de Kleijn et al 2012	GSE35590	2ng/kg of body weight 4h (in vivo)	Affymetrix Human Exon 1.0 ST Array	4	444 up<br>435 down
de Kleijn et al 2012	GSE35590	2ng/kg of body weight 6h (in vivo)	Affymetrix Human Exon 1.0 ST Array	4	392 up<br>446 down
de Kleijn et al 2013	GSE42358	2ng/kg of body weight 4h (in vivo) $CD16^{dim}/CD62L^{bright}$ NPs	Affymetrix Human Exon 1.0 ST Array	4	216 up<br>591 down
de Kleijn et al 2013	GSE42358	2ng/kg of body weight 4h (in vivo) $CD16^{bright}/CD62L^{bright}$ NPs	Affymetrix Human Exon 1.0 ST Array	4	386 up<br>372 down
de Kleijn et al 2013	GSE42358	2ng/kg of body weight 4h (in vivo) $CD16^{bright}/CD62L^{dim}$ NPs	Affymetrix Human Exon 1.0 ST Array	4	468 up<br>333 down
Zhang et al	N/A	10, 20, 30, 60, 120 min 10ng/ml (in vitro)	HG_U95A version 2 GeneChip arrays	3	<u>1.8-fold:</u><br>227 up<br>133 down
Coldren et al	GSE2322	100 ng/ml 60 min (in vitro)	Affymetrix Human Genome U133A Array	12	124 up<br>128 down
Fessler et al	N/A	100 ng/ml 4h (in vitro)	Affymetrix HuGene 6800FL Genechips	3	99 up (3-fold)<br>53 down (4-fold)
Coldren et al	GSE2322	100 ng/ml 60 min (in vivo)	Affymetrix Human Genome U133A Array	12	38 up<br>18 down
Silva et al	GSE3037	100 ng/ml 60 min (in vitro)	Affymetrix Human Genome U133A Array	8 (patients with sepsis-induced acute lung injury)	46 up<br>3 down
Malcolm et al	N/A	100 ng/ml 4h (in vitro)	cDNA array	3	25 up<br>11 down
**Mouse**					
Khatib-Massalha et al	GSE143978	50 µg intraperitoneal injection 4h (in vivo) BM NPs	Illumina NextSeq 500	3 Ctrl<br>5 LPS	1405 up<br>1268 down
Hutchins et al	GSE55385	100ng/ml 4h (in vitro) BM NPs	Illumina HiSeq 2000	2	871 up<br>869 down

Table S1. Datasets included in the meta-analysis for LPS-stimulated neutrophils. All datasets included in the analysis with associated publication reference, GEO accession number, dose and time of LPS treatment, platform, and number of biological replicates and 2-fold regulated genes. References are listed on the next page. BM NPs = bone marrow neutrophils.

References for Table S1.

Kotz KT, Xiao W, Miller-Graziano C, Qian WJ, Russom A, Warner EA, Moldawer LL, De A, Bankey PE, Petritis BO, Camp DG. Clinical microfluidics for neutrophil genomics and proteomics. Nature medicine. 2010 Sep;16(9):1042-7.

Khaenam P, Rinchai D, Altman MC, Chiche L, Buddhisa S, Kewcharoenwong C, Suwannasaen D, Mason M, Whalen E, Presnell S, Susaengrat W. A transcriptomic reporter assay employing neutrophils to measure immunogenic activity of septic patients' plasma. Journal of translational medicine. 2014 Dec;12(1):1-4.

De Kleijn S, Kox M, Sama IE, Pillay J, Van Diepen A, Huijnen MA, Van der Hoeven JG, Ferwerda G, Hermans PW, Pickkers P. Transcriptome kinetics of circulating neutrophils during human experimental endotoxemia. PLoS One. 2012 Jun 5;7(6):e38255.

de Kleijn S, Langereis JD, Leentjens J, Kox M, Netea MG, Koenderman L, Ferwerda G, Pickkers P, Hermans PW. IFN-γ-stimulated neutrophils suppress lymphocyte proliferation through expression of PD-L1. PloS one. 2013 Aug 28;8(8):e72249.

Zhang X, Kluger Y, Nakayama Y, Poddar R, Whitney C, DeTora A, Weissman SM, Newburger PE. Gene expression in mature neutrophils: early responses to inflammatory stimuli. Journal of leukocyte biology. 2004 Feb;75(2):358-72.

Coldren CD, Nick JA, Poch KR, Woolum MD, Fouty BW, O'Brien JM, Gruber MP, Zamora MR, Svetkauskaite D, Richter DA, He Q. Functional and genomic changes induced by alveolar transmigration in human neutrophils. American Journal of Physiology-Lung Cellular and Molecular Physiology. 2006 Dec;291(6):L1267-76.

Fessler MB, Malcolm KC, Duncan MW, Worthen GS. A genomic and proteomic analysis of activation of the human neutrophil by lipopolysaccharide and its mediation by p38 mitogen-activated protein kinase. Journal of Biological Chemistry. 2002 Aug 30;277(35):31291-302.

Coldren CD, Nick JA, Poch KR, Woolum MD, Fouty BW, O'Brien JM, Gruber MP, Zamora MR, Svetkauskaite D, Richter DA, He Q. Functional and genomic changes induced by alveolar transmigration in human neutrophils. American Journal of Physiology-Lung Cellular and Molecular Physiology. 2006 Dec;291(6):L1267-76.

Silva E, Arcaroli J, He Q, Svetkauskaite D, Coldren C, Nick JA, Poch K, Park JS, Banerjee A, Abraham E. HMGB1 and LPS induce distinct patterns of gene expression and activation in neutrophils from patients with sepsis-induced acute lung injury. Intensive care medicine. 2007 Oct;33(10):1829-39.

Malcolm KC, Arndt PG, Manos EJ, Jones DA, Worthen GS. Microarray analysis of lipopolysaccharide-treated human neutrophils. American Journal of Physiology-Lung Cellular and Molecular Physiology. 2003 Apr 1;284(4):L663-70.

Khatib-Massalha E, Bhattacharya S, Massalha H, Biram A, Golan K, Kollet O, Kumari A, Avemaria F, Petrovich-Kopitman E, Gur-Cohen S, Itkin T. Lactate released by inflammatory bone marrow neutrophils induces their mobilization via endothelial GPR81 signaling. Nature communications. 2020 Jul 15;11(1):1-8.

Hutchins AP, Takahashi Y, Miranda-Saavedra D. Genomic analysis of LPS-stimulated myeloid cells identifies a common pro-inflammatory response but divergent IL-10 anti-inflammatory responses. Scientific reports. 2015 Mar 13;5(1):1-2.

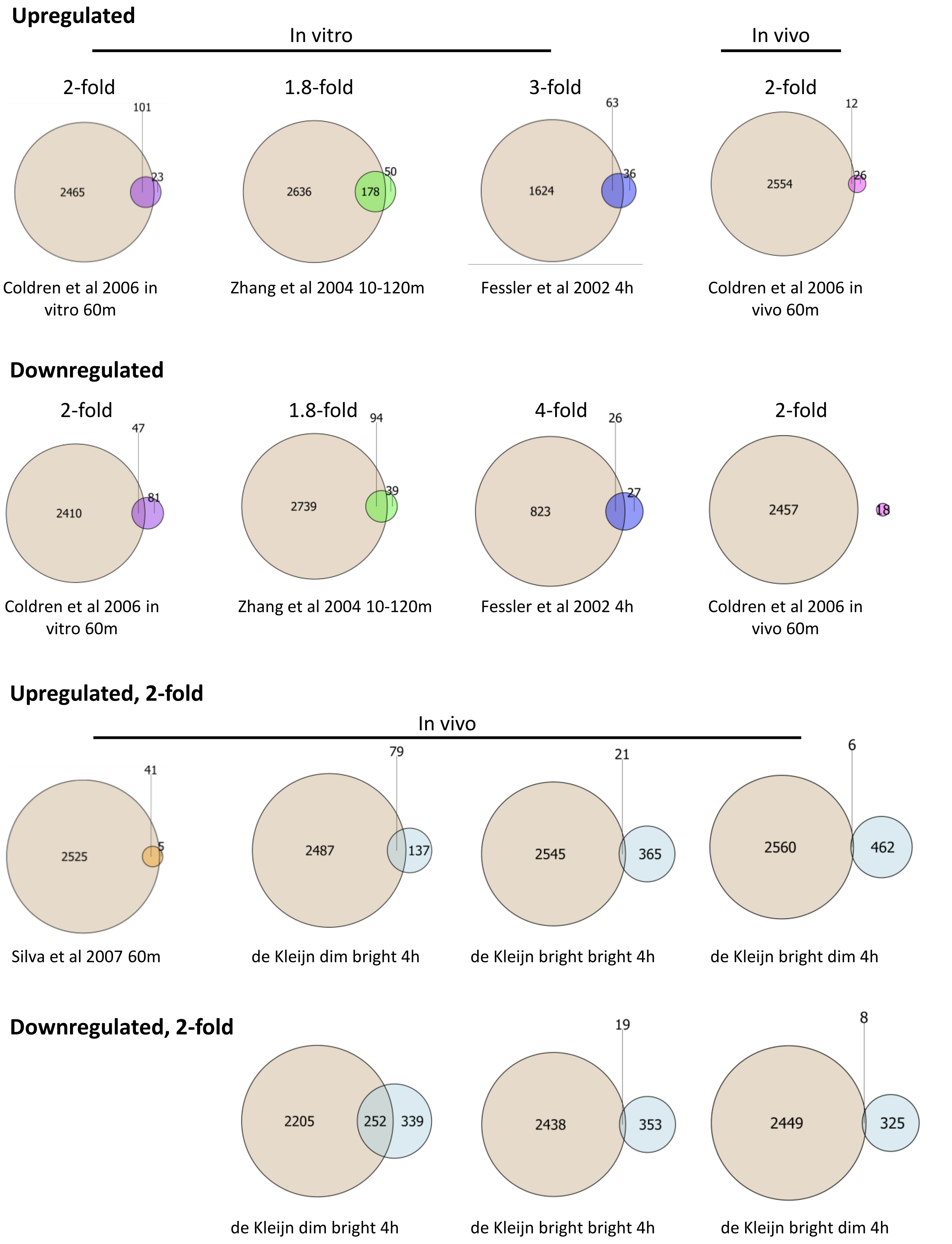

Figure S1. Venn diagrams illustrating partial overlap in genes regulated 2-fold in our dataset and other in vitro and in vivo datasets from LPS-stimulated neutrophils. Tan colour represents our 6h data and other colours represent previously published datasets, as indicated in the figure.

Upregulated, 2-fold **Downregulated, 2-fold**

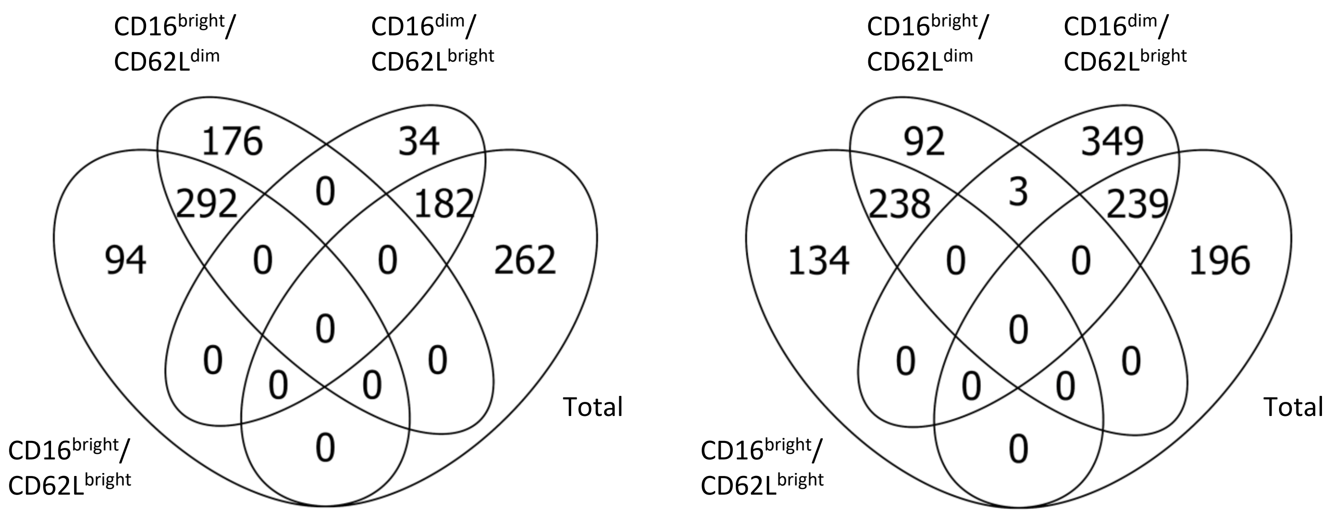

de Kleijn 4h

Figure S2. Venn diagrams of 3 sorted subpopulations and total neutrophils from 4h LPS-stimulated neutrophil datasets from de Kleijn et al 2013 and 2012, respectively. Little to no overlap is illustrated between the subpopulations and total cells, except for a partial overlap observed for total and CD16dim/CD62Lbright cells.

Examples of PCA plots

Khatib-Massalha et al 2020 (mouse)
https://www.ncbi.nlm.nih.gov/geo/query/acc.cgi?acc=GSE143978

NCBI GEO Gene Expression Omnibus

COVID-19 is an emerging, rapidly evolving situation.
Get the latest public health information from CDC: https://www.coronavirus.gov .
Get the latest research from NIH: https://www.nih.gov/coronavirus.
Find NCBI SARS-CoV-2 literature, sequence, and clinical content: https://www.ncbi.nlm.nih.gov/sars-cov-2/.

HOME | SEARCH | SITE MAP — GEO Publications | FAQ | MIAME | Email GEO

NCBI > GEO > Accession Display — Not logged in | Login

Scope: Self — Format: HTML — Amount: Quick — GEO accession: GSE143978 — GO

Series GSE143978	Query DataSets for GSE143978
Status	Public on Jun 03, 2020
Title	Lactate released by inflammatory bone marrow neutrophils induces their mobilization via endothelial GPR81 signaling
Organism	Mus musculus
Experiment type	Expression profiling by high throughput sequencing
Summary	We found different expression between glycolytic enzymes and TCA pathway in bone marrow neutrophils following LPS treatment, using RNAseq. This enable us to uncover the important role of glycolysis in activated neutrophils and the effect of HIF-1alpha on this pathway

Scroll to the bottom of the page and click on "**SRA Run Selector**"

Download family	Format
SOFT formatted family file(s)	SOFT
MINiML formatted family file(s)	MINiML
Series Matrix File(s)	TXT

Supplementary file	Size	Download	File type/resource
GSE143978_mcSCRBseq_samples_barcodes.txt.gz	222 b	(ftp)(http)	TXT
GSE143978_raw_UMI_counts.txt.gz	243.0 Kb	(ftp)(http)	TXT

SRA Run Selector
Raw data are available in SRA
Processed data are available on Series record

Vehicle
LPS-treated

Run	BioSample	Bases	Bytes	Experiment	GEO_Accession	Sample Name	sex	source_name	Treatment
SRR10918622	SAMN13895429	465.17 M	197.57 Mb	SRX7585923	GSM4277558	GSM4277558	male	WT_PBS_male	PBS
SRR10918623	SAMN13895428	1.20 G	512.61 Mb	SRX7585924	GSM4277559	GSM4277559	male	WT_LPS_male	LPS
SRR10918624	SAMN13895427	1.44 G	614.07 Mb	SRX7585925	GSM4277560	GSM4277560	male	WT_PBS_male	PBS
SRR10918625	SAMN13895426	87.81 M	36.97 Mb	SRX7585926	GSM4277561	GSM4277561	male	WT_LPS_male	LPS
SRR10918626	SAMN13895425	822.33 M	347.05 Mb	SRX7585927	GSM4277562	GSM4277562	male	WT_LPS_male	LPS
SRR10918627	SAMN13895424	1.07 G	452.72 Mb	SRX7585928	GSM4277563	GSM4277563	female	WT_LPS_female	LPS
SRR10918628	SAMN13895423	1.36 G	573.44 Mb	SRX7585929	GSM4277564	GSM4277564	female	WT_LPS_female	LPS
SRR10918629	SAMN13895422	1.19 G	505.02 Mb	SRX7585930	GSM4277565	GSM4277565	male	WT_PBS_male	PBS

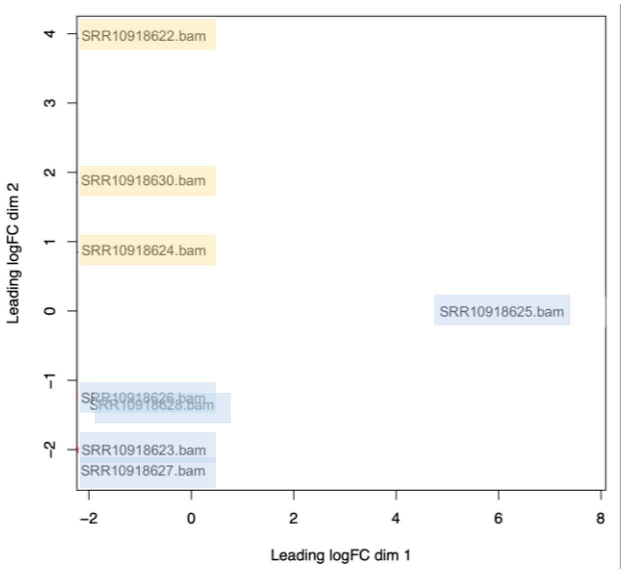

Our data (human)

Vehicle
LPS-treated

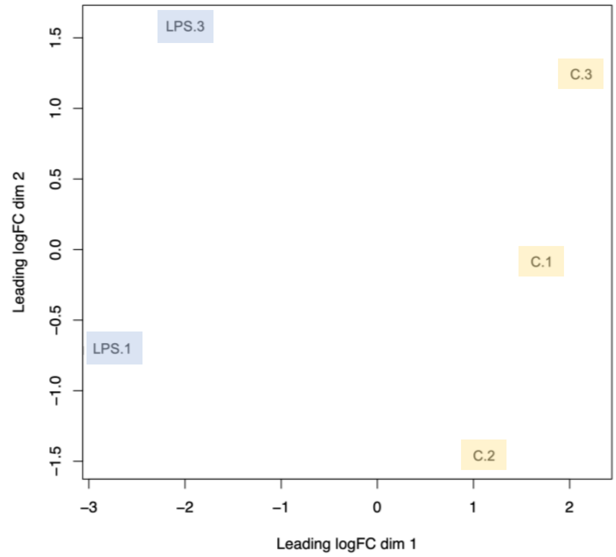

Figure S3. Example of PCA plot in mouse and our human datasets. Similarity between replicates in each group is confirmed by PCA analysis.

Examples of boxplots for raw and normalized expression values

Kotz et al 2010
https://www.ncbi.nlm.nih.gov/geo/query/acc.cgi?acc=GSE22103

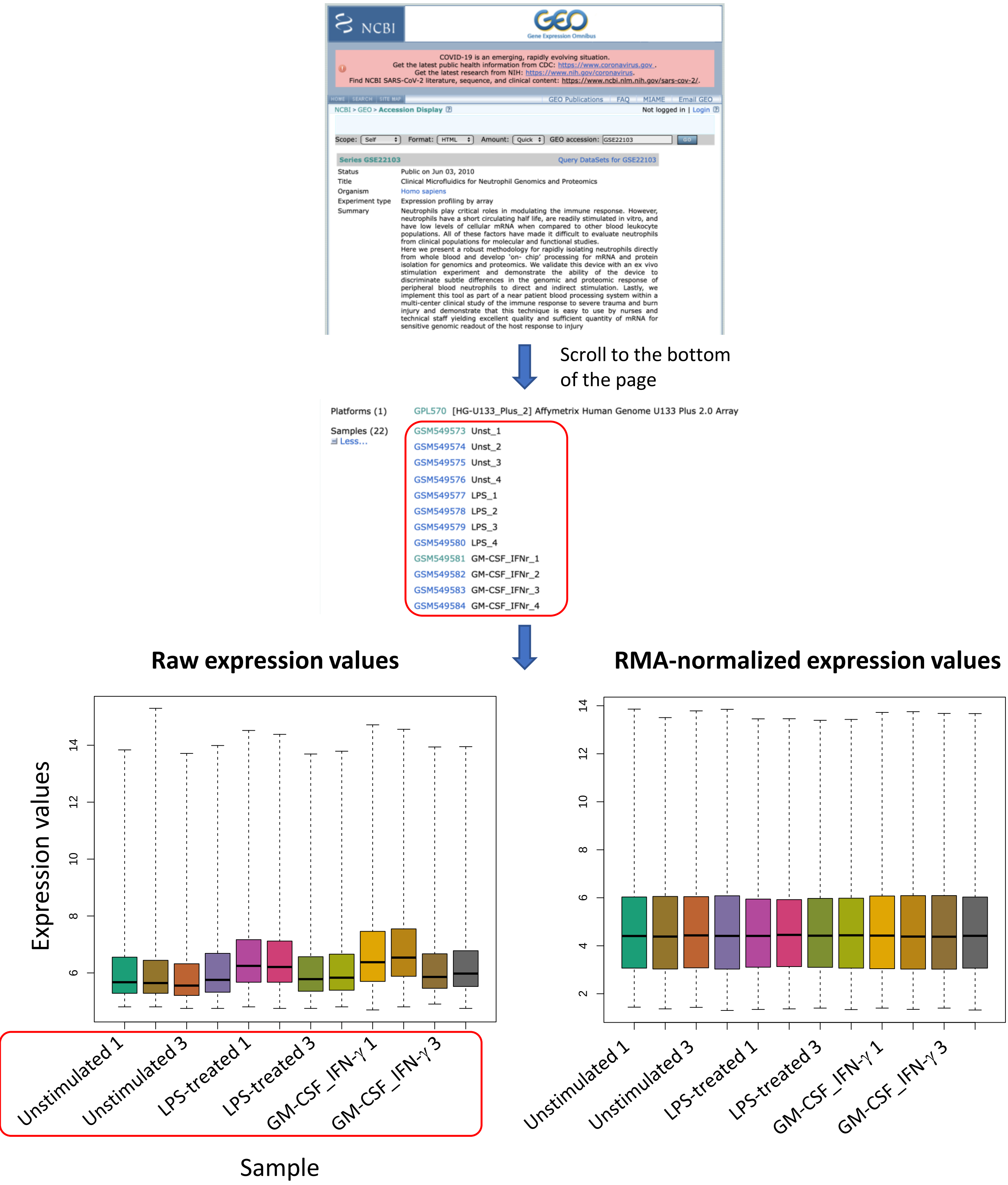

Figure S4. Examples of boxplots for raw and normalized expression values in human microarray datasets. To identify potential outliers, signal distribution of the raw and normalized data was viewed using boxplots. Samples were comparable following background correction and RMA normalization.

Western blot Fig 5D: Neutrophils **Western blot Fig 7A: HEK293**

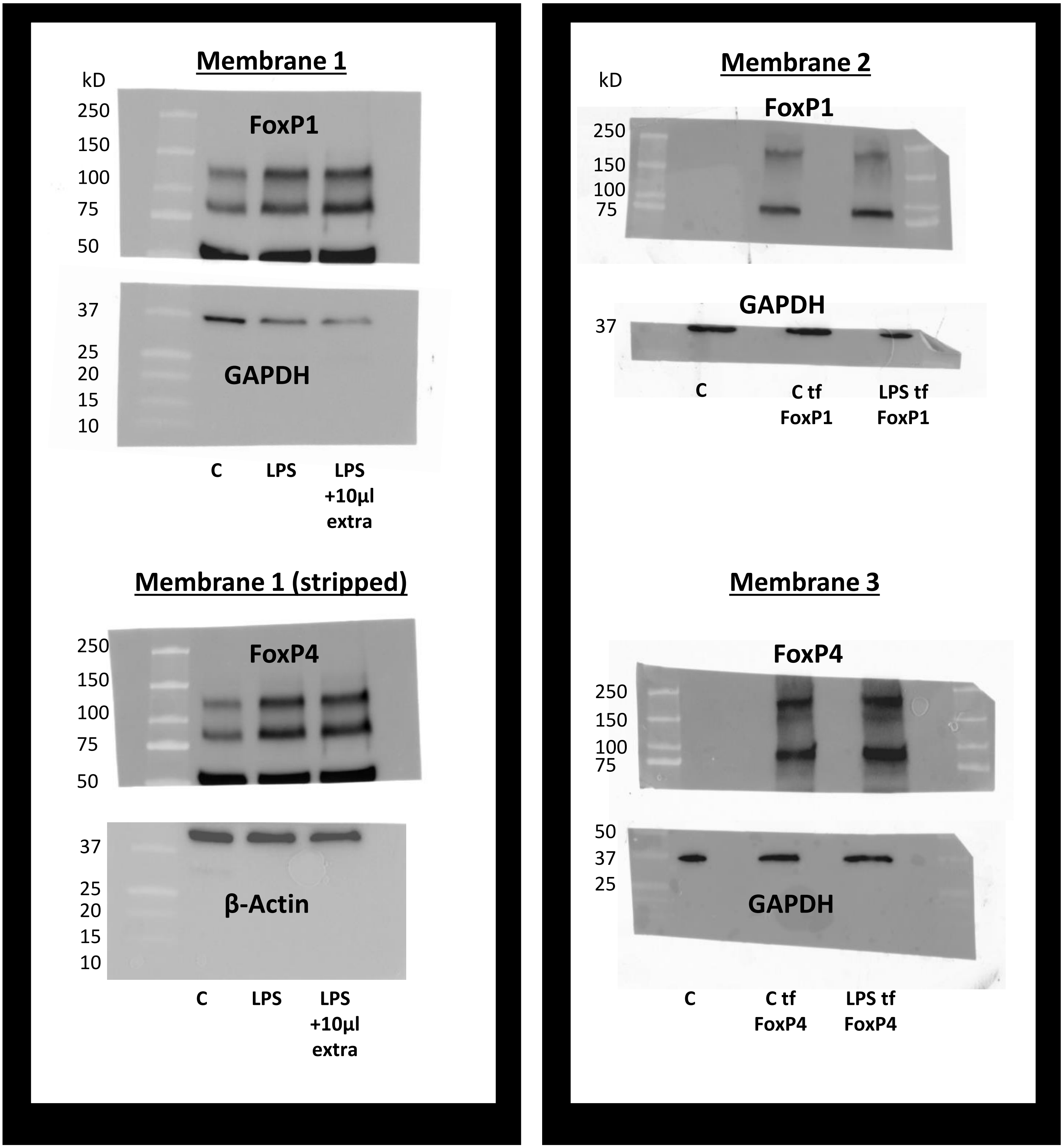

Figure S5. Full western blots of FoxP1 and FoxP4 probed neutrophils and HEK293 cells. The ~85 kDa band corresponds to the full-length FoxP1 and FoxP4 protein as described in (50, 70-73). Tf = transfected HEK293 cells.

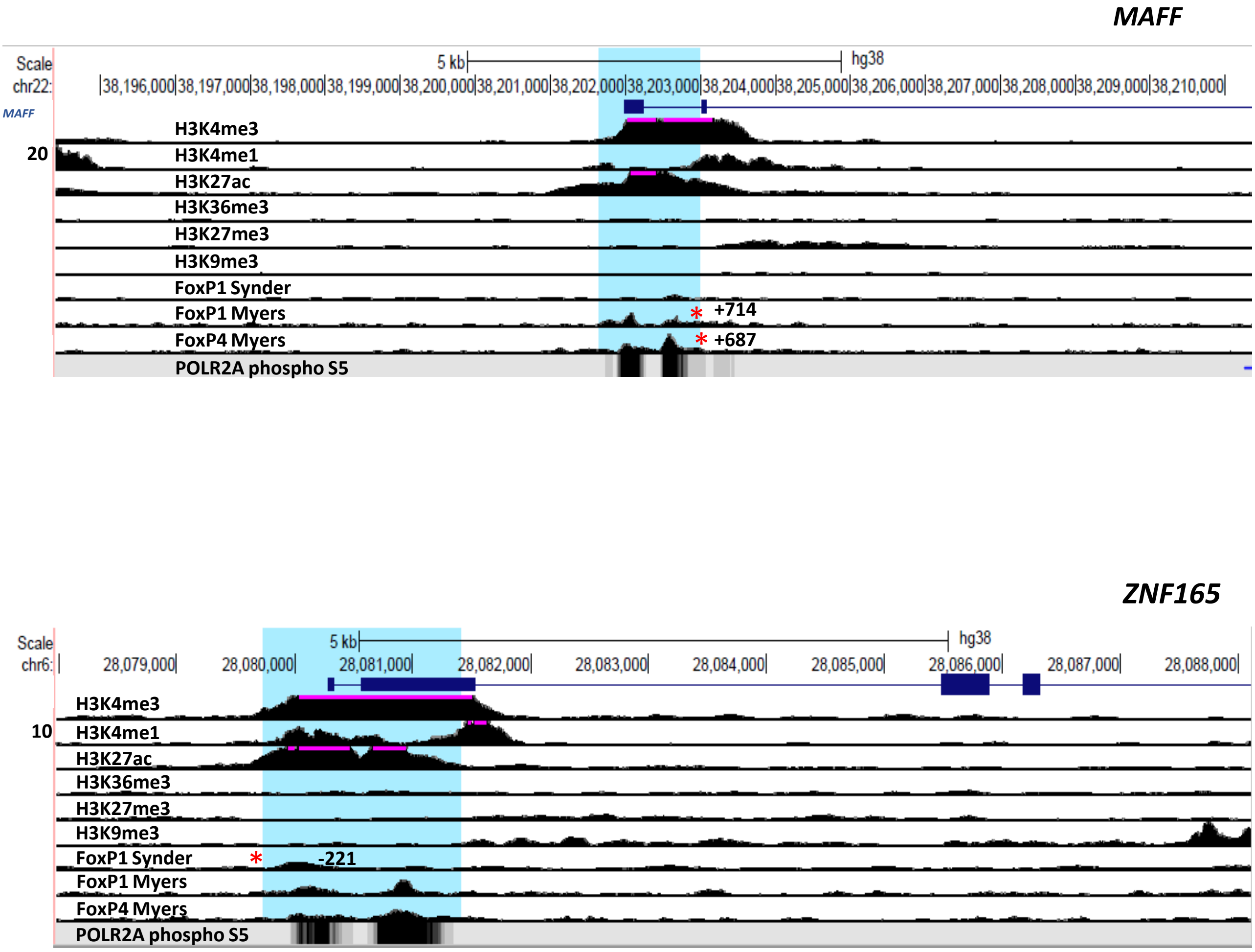

Figure S6. UCSC browser images of FOXP1 and FOXP4 ChIPseq tracks for the MAFF and ZNF165 loci. The areas surrounding the FOXP1 and/or FOXP4 binding sites of genes are shown in blue. Red asterisks represent ChIPseq peaks that contain a consensus motif. Binding sites correspond to regions of enhancer function in neutrophils.

ENCODE ChIPseq experiment	Target	Platform	Assembly	Lab / Reference	Cell Type
N/A	FOXP1	SOLiD/AB sequencer	hg19	van Boxtel et al 2013	Colon cancer cell line (DLD1)
N/A	FOXP1	Illumina Genome Analyzer II	hg19	Gabut et al 2011	Embryonic stem cells
ENCSR029LBT	FOXP1	Illumina HiSeq 2000	hg38, hg19	Michael Snyder Lab	HepG2
ENCSR369YUK	FOXP1	Illumina NextSeq 500	hg38, hg19	Richard Myers HAIB	HepG2 genetically modified with CRISPR
ENCSR232LLP	FOXP4	Illumina NextSeq 500	hg38	Richard Myers HAIB	HepG2 genetically modified with CRISPR
ENCSR393SYU - ENCFF628GHA	H3K4me3	Illumina HiSeq 2500	hg38, hg19	Bradley Bernstein, Broad	Homo sapiens neutrophil
ENCSR586POT - ENCFF897XDW	H3K4me1	Illumina Genome Analyzer IIx	hg38, hg19	Bradley Bernstein, Broad	Homo sapiens neutrophil male
ENCSR267YXV - ENCFF909JRL	H3K27ac	Illumina HiSeq 2500	hg38, hg19	Bradley Bernstein, Broad	Homo sapiens neutrophil
ENCSR373WCB - ENCFF356ZPT	H3K36me3	Illumina HiSeq 2500	hg38, hg19	Bradley Bernstein, Broad	Homo sapiens neutrophil
ENCSR058FCG - ENCFF981CZJ	H3K27me3	Illumina HiSeq 2500	hg38, hg19	Bradley Bernstein, Broad	Homo sapiens neutrophil
ENCSR437MHW - ENCFF777GYB	H3K9me3	Illumina Genome Analyzer IIx	hg38, hg19	Bradley Bernstein, Broad	Homo sapiens neutrophil male
ENCSR960ALP - ENCFF852BHO	POLR2A phosphoS5	Not specified	hg38	Bill Noble, UW	Homo sapiens neutrophil male
ENCSR208XPO - ENCFF514ZFZ	POLR2A phosphoS5	Not specified	hg38	Bill Noble, UW	Homo sapiens neutrophil

Table S2. ChIPseq datasets included in this study. All datasets included in the study with associated ENCODE experiment ID (if applicable), target, platform, assembly, lab or publication reference and cell type.

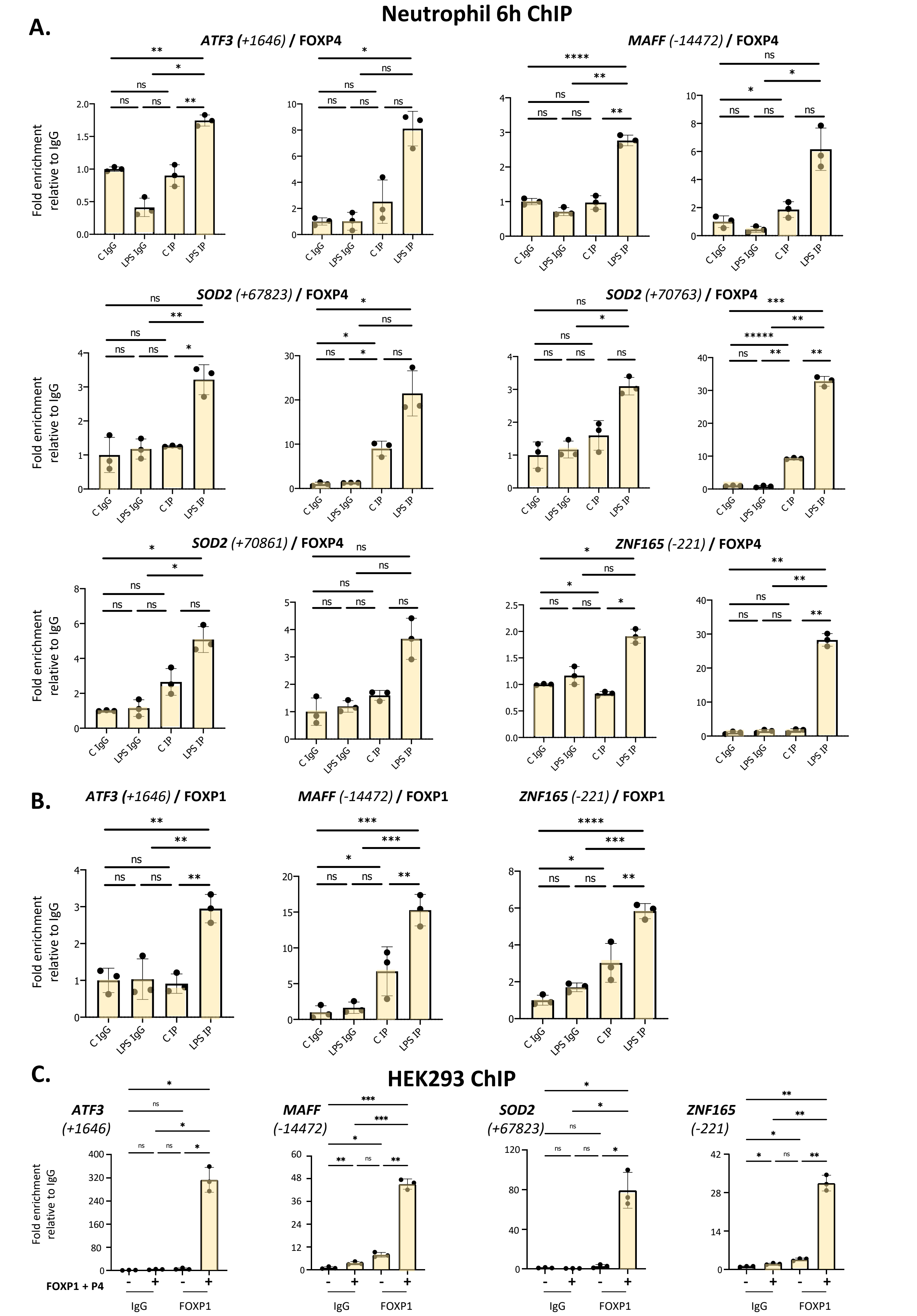

Figure S7. (figure legend on next page)

Figure S7. Enhanced binding of FOXP4 and FOXP1 to network gene motifs in individual isolates of LPS-challenged neutrophils and HEK293 cells. A. Analysis of the association of FOXP4 with up- and downstream regulatory regions of ATF3, SOD2, MAFF and ZNF165 by ChIP assay in individual isolates of neutrophils treated with or without LPS for 6h (not including the representative data). B. Analysis of the association of FOXP1 with up- and downstream regulatory regions of ATF3, SOD2, MAFF and ZNF165 by ChIP assay in one isolate of neutrophils treated with or without LPS for 6h. C. Analysis of the association of FOXP1 with up- and downstream regulatory regions of ATF3, MAFF, SOD2 and ZNF165 by ChIP assay in HEK293 cells transfected with and without FOXP1 and FOXP4 expression vectors. Data representative of 2 or 3 biological replicates. All graphics are mean ± SD from 3 technical replicates from a representative sample.*P ≤ 0.05, **P ≤ 0.01, ***P ≤ 0.001, ****P ≤ 0.0001 and ns ≥0.05 as assessed by one-way ANOVAs followed by Tukey's post hoc test for multiple comparisons. ChIP values are normalized to input for each condition and expressed as a fold relative to non-specific IgG control.

Study	GEO accession	Dose and time of treatment	Platform	Number of biological replicates	Number of regulated genes (2-fold)
**Human**					
Our data	GSE221866	1 µg/ml 6h (in vitro) LPS	Illumina NovaSeq 6000	3	2565 up<br>2457 down
Prince et al 2017	GSE94923	1 mM 4h (in vitro) N6/8-AHA (PKA agonist)	Affymetrix Human Genome U133 Plus 2.0 Array	3	1241 up<br>1879 down
Kotz et al 2010	GSE22103	20 ng/mL GM-CSF and 100 IU/ml IFN-γ 16h	Affymetrix Human Genome U133 Plus 2.0 Array	4	2799 up<br>2271 down
Greenberg et al 2015	GSE55849	*Granulibacter bethesdensis* 24h	Affymetrix Human Genome U133 Plus 2.0 Array	4	1791 up<br>1902 down
Wright et al 2010	GSE20151	*Fusobacterium nucleatum* MOI 1:300 3h	Affymetrix Human Genome U133A Array	2	249 up<br>41 down

References for Table S3.

Prince LR, Prosseda SD, Higgins K, Carlring J, Prestwich EC, Ogryzko NV, Rahman A, Basran A, Falciani F, Taylor P, Renshaw SA. NR4A orphan nuclear receptor family members, NR4A2 and NR4A3, regulate neutrophil number and survival. Blood, The Journal of the American Society of Hematology. 2017 Aug 24;130(8):1014-25.

Kotz KT, Xiao W, Miller-Graziano C, Qian WJ, Russom A, Warner EA, Moldawer LL, De A, Bankey PE, Petritis BO, Camp DG. Clinical microfluidics for neutrophil genomics and proteomics. Nature medicine. 2010 Sep;16(9):1042-7.

Greenberg DE, Sturdevant DE, Marshall-Batty KR, Chu J, Pettinato AM, Virtaneva K, Lane J, Geller BL, Porcella SF, Gallin JI, Holland SM. Simultaneous host-pathogen transcriptome analysis during Granulibacter bethesdensis infection of neutrophils from healthy subjects and patients with chronic granulomatous disease. Infection and immunity. 2015 Nov;83(11):4277-92.

Wright HJ, Chapple IL, Matthews JB, Cooper PR. Fusobacterium nucleatum regulation of neutrophil transcription. Journal of periodontal research. 2011 Feb;46(1):1-2.

Table S3. Datasets included in the meta-analysis for neutrophils challenged with various inflammatory signals. All datasets included in the analysis with associated publication reference, GEO accession number, dose and time of LPS treatment, platform, and number of biological replicates and 2-fold regulated genes.

Primers qPCR

Targeted gene	Forward sequence	Reverse sequence
**Primers RT/qPCR**		
ATF3	5’-AAGAGCTGAGGTTTGCCATC-3’	5’-TCTTTCTCGTCGCCTCTTTT-3’
BCL2A1	5’-TTACAGGCTGGCTCAGGACT-3’	5’-AGCACTCTGGACGTTTTGCT-3’
CAMP	5’-GACAGTGACCCTCAACCAGG-3’	5’-AGGGCACACACTAGGACTCT-3’
CCL20	5’-TTTATTGTGGGCTTCACACG-3’	5’-ATTTGCGCACACAGACAACT-3’
CD40	5’-CTTCTTCACAGGTGCAGATG-3’	5’-GTTCACTGAAACGGAATGCC-3’
CLEC4E	5’-AATTTCACAGAGCTCTCCTGCT-3’	5’-TGCTCCTCCTGTGAGTTGATAA-3’
CLEC7A	5’-CACTAAATTCCTGGGATGGAAG-3’	5’-GTTTTCTTGGGTAGCTGTGGTT-3’
CSF3	5’-CAAGTGCTTAGAGCAAGTGAGG -3’	5’-TAGAGGAAAAGGCCGCTATG-3’
CXCL3	5’-AAGTGTGAATGTAAGGTCCCC-3’	5’-GTGCTCCCCTTGTTCAGTATC-3’
CXCL8	5’-CTGCGCCAACACAGAAATTA-3’	5’-ACTTCTCCACAACCCTCTGC-3’
CXCR2	5’-AACATGGAGAGTGACAGCTTTG-3’	5’-CAGGGCATAGATAATGACCACA-3’
FCER1G	5’-AGCTCTGCTATATCCTGGATGC-3’	5’-GTTATAGCTGCCTTTCGCACTT-3’
FOXP1	5’-ATGCGCTGGACGATAGAAGT-3’	5’-GCGGACTTGGAGAGAGTGAC-3’
FOXP4	5’-CACCAGGATGTTCGCCTATT-3’	5’-TTCTGATACTCCCGCTCGTC-3’
ICAM1	5’-CCGAGCTCAAGTGTCTAAAGGA-3’	5’-CGGTTATAGAGGTACGTGCTGA-3’
IFNGR2	5’-GTCGGGCATTTAAGCAACAT-3’	5’-AATGTTCCCACGGAGATCAG-3’
IL1B	5’-GGACAAGCTGAGGAAGATGC-3’	5’-TCGTTATCCCATGTGTCGAA-3’
IL36G	5’-ATCACATGCAAGTATCCAGAGG-3’	5’-ACGGTAGAAAAGGAAGGGTTTC-3’
LIF	5’-ATACGCCACCCATGTCACAA-3’	5’-GCCAAGGTACACGACTATGC-3’
MAFF	5’-ATCCCCTATCCAGCAAAGCTC-3’	5’-TTGAGCCGTGTCACCTCCTC-3’
NOD2	5’CTCCATGGCTAAGCTCCTTG-3’	5’-CACACTGCCAATGTTGTTCC-3’
NR4A3	5’-TGCCCAGTAGACAAGAGACG-3’	5’-ACGACCTCTCCTCCCTTTCA-3’
P2RX7	5’-CAGTGTCCCATTTTCCGACT-3’	5’-AGGGTACAAGGACACGTTGG-3’
SERPINB2	5’-ATGAAATTGCCGATGTGTCCAC-3’	5’-CTTTGCTGGTCCACTTGTTGAG-3’
SOD2	5’-TCAATAAGGAACGGGGACAC-3’	5’-AATCCCCAGCAGTGGAATAA-3’
TNF	5’-ACAAGCCTGTAGCCCATGTT-3’	5’-GAGGTACAGGCCCTCTGATG-3’
TNFAIP3	5’-GCACACTGTGTTTCATCGAGTA-3’	5’-AGTTGCTCTTCTGTCCTTTTGG-3’
ZC2HC1C	5’-GAAGTCGTGCAGTACAGGTGAG-3’	5’-GCAAGAACAGTACCAACCACAG-3’
ZNF165	5’-TAAGCCACGATGGATGTGAG-3’	5’-AGCAAGTTTTGGGCTCTTGA-3’
18S	5’-GCAATTATTCCCCATGAACG-3’	5’-GGGACTTAATCAACGCAAGC-3’
**Primers ChIP-qPCR**		
ATF3+1646	5’-TCACTAGCGTGTTAGCAAGGTT-3’	5’-TGAACTCCCACACAACTGAGG-3’
BCL2A1-9259	5’-GCATCTGACCAGTGACTTTGAT-3’	5’-AAGGAGCATAAGGGCATAAGG-3’
MAFF-14472	5’-AGCAGCAGTGGGCAAACTAT-3’	5’-GTGGGCCATGAGGTATCTGT-3’
SOD2+67823	5’-CACCTGAACTTGGCTTCACA-3’	5’-TTCCTTTTTCACCCCAACAC-3’
ZNF165-221	5’-CATTTTCCTCGTCTCCTTGC-3’	5’-GGTCACCAGGGGAACAGAT-3’

Table S4. Primer sequences for RT/qPCR and ChIP-qPCR.

List of abbreviations

Abbreviation	Full name
NF-κB	Nuclear factor-kappa B
RNAseq	RNA sequencing
LPS	Lipopolysaccharide
RT/qPCR	Reverse transcription followed by quantitative polymerase chain reaction
GEO	Gene Expression Omnibus data repository
LIMMA	Linear Models for Microarray Data R package
QC	Quality control
FOXL2	Forkhead box transcription factor FOXL2
KGN	human ovarian granulosa-like tumour cell line
FOXP1	Forkhead box transcription factor FOXP1
FOXP4	Forkhead box transcription factor FOXP4
FOX	Forkhead box
ENCODE	Encyclopedia of DNA Elements
TRAP	Transcription factor Affinity Prediction web tool
ChIP	Chromatin Immunoprecipitation
ChIPseq	Chromatin Immunoprecipitation sequencing
TSS	Transcription start site
UCSC	University of California Santa Cruz Genome Browser
HEK293	Human embryonic kidney 293 cell line
N6/8-AHA	protein kinase A agonist pair 8-AHA-cAMP and N6-MB-cAMP
GM-CSF	Granulocyte-macrophage colony-stimulating factor cytokine
IFN-γ	Interferon-gamma cytokine
IPA	Ingenuity Pathway Analysis (QIAGEN)
FACS	Fluorescence-activated cell sorting
HL-60	Human leukemia cell line

Table S5. List of abbreviations used in the main text.

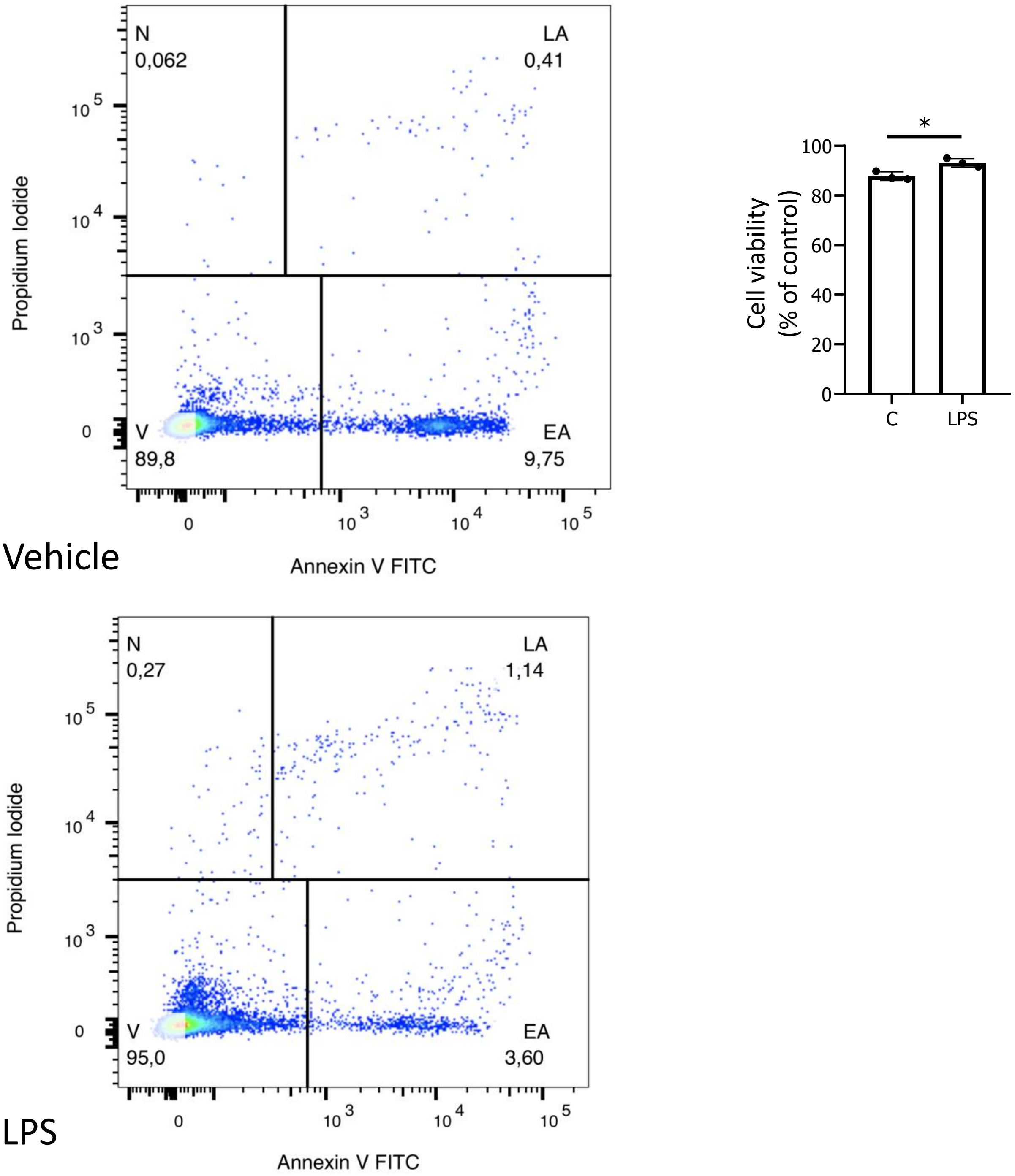

Figure S8. Viability of neutrophils treated for 6h with vehicle and LPS. Cells were gated for singlets, and debris as well as erythrocytes were excluded using the forward and side scatter plot. Viable neutrophils are identified as being negative for both annexin V and propidium iodide. N = non-viable, LA = late-apoptotic, EA = early-apoptotic, V = viable. The data shown is representative of 2 independent experiments. *P ≤ 0.05 by Student's t-test.
